# Cortical thinning in subclinical hypothyroidism: structural and functional brain changes underlying cognitive impairment

**DOI:** 10.1186/s12902-026-02210-4

**Published:** 2026-03-04

**Authors:** Shuai Zhao, Jindan Wu, Xiaomei Liu, Yi Xia, Xumiao Wang, Zhilu Chen, Rui Yan, Hao Tang, Qing Lu, Zhijian Yao

**Affiliations:** 1 Department of Psychiatry, The Affiliated Psychological Hospital of Anhui Medical University, Hefei, China; 2 Hefei Fourth People’s Hospital, Hefei, China; 3 Anhui Mental Health Center, Hefei, China; 4 Anhui Clinical Research Center for Mental Disorders, Hefei, China; 5 Department of Psychiatry, The Affiliated Brain Hospital of Nanjing Medical University, Nanjing, 210029 People’s Republic of China; 6 School of Biological Sciences & Medical Engineering, Southeast University, Nanjing, 210096 People’s Republic of China; 7 Key Laboratory of Ministry of Education, Child Development and Learning Science, Nanjing, China; 8 Nanjing Brain Hospital, Medical School of Nanjing University, Nanjing, China; 9 Department of Endocrinology, Nanjing First Hospital, Nanjing Medical University, Nanjing, China

**Keywords:** Subclinical hypothyroidism, Cortex thickness, fMRI, Cognition

## Abstract

Subclinical Hypothyroidism (SHypo), a condition that characteristically presents with normal Thyroid Hormone (TH) levels coexisting with elevated Thyroid-Stimulating Hormone (TSH) levels, has been associated with cognitive impairment. Nonetheless, its neurobiological effects remain somewhat unclear, forming the basis of this study. Herein, using a multimodal neuroimaging approach, we explored cortical thinning and disrupted Functional Connectivity (FC) in SHypo patients—particularly focusing on their relationship with cognitive deficits. Brain areas such as the Prefrontal Cortex (PFC) and Cingulate Gyrus (CG) exhibited substantial cortical thinning. Additionally, a marked decrease in FC was observed between the CG and regions associated with visuospatial processing. Furthermore, serum-free thyroxine (FT4) levels correlated negatively with Cortex Thickness (CT) of the right cuneus. Notably, FC also correlated positively with neuropsychological tests. Besides highlighting the potential for early neural alterations in SHypo, these findings also underscore the significance of early detection and intervention to mitigate cognitive decline.

## Clinical trial number

Not applicable.

## Background

Thyroid Hormones (THs) play crucial roles in brain development and function, regulating essential processes including synaptogenesis, myelination, and cerebral metabolism [1–4]. Subclinical Hypothyroidism (SHypo) is a condition characterized by elevated serum Thyroid-Stimulating Hormone (TSH) levels with normal free thyroxine (FT4) and free triiodothyronine (FT3) concentrations [5].

SHypo affects 4–10% of the general adult population, with prevalence increasing with age and a marked female predominance (female-to-male ratio of 4–9:1) [6, 7]. Diagnostic criteria remain controversial, as different guidelines recommend varying TSH thresholds (ranging from 2.5 to 10 mIU/L) for defining the upper limit of normal [8, 9]. This diagnostic ambiguity extends to treatment, with ongoing debate over the necessity and efficacy of levothyroxine therapy to prevent potential complications, such as cognitive decline [10].

This hormonal imbalance can disrupt key brain processes and has been associated with cognitive deficits [11, 12]. Despite its high prevalence [7, 13], the clinical significance of SHypo—particularly its neurological implications—remains debated. While some studies linked SHypo with impaired memory and Executive Function (EF), with one study reporting a significant reduction in cognitive control task accuracy (Cohen’s *d* = 1.8, *p* < 0.01) and prolonged reaction times (Cohen’s *d* = 1.6, *p* < 0.01) [14], others reported no significant neuropsychological differences relative to euthyroid individuals [15], implying a heterogeneous neural compensation or undetected structural-functional alterations. Furthermore, previous research has focused mainly on overt hypothyroidism, often overlooking the subtle cortical changes associated with SHypo, thus yielding limited and inconsistent neuroimaging evidence [16–19]. This underscores the need for more sensitive, region-specific approaches to determine whether SHypo is accompanied by measurable brain alterations.

Cortical thinning (CT) mapping provides a sensitive marker of regional cortical integrity. It has been widely applied to detect subtle structural changes across neuropsychiatric and endocrine-related conditions [20, 21]. In parallel, resting-state functional magnetic resonance imaging (rs-fMRI) enables characterization of intrinsic functional connectivity (FC), offering complementary information about interregional communication and network organization. Recent rs-fMRI studies in SHypo have reported alterations in resting-state networks implicated in cognitive control and memory-related processing [14, 22], suggesting that functional measures may capture early or subtle neural effects of subclinical thyroid dysfunction. Nevertheless, how such functional alterations relate to cortical morphology and cognitive performance in SHypo remains insufficiently understood.

Therefore, in the present study, we employed a multimodal neuroimaging design combining CT analysis and rs-fMRI–based FC assessment to investigate structural and functional brain alterations in SHypo. We aimed to (i) identify regions showing CT differences between SHypo patients and healthy controls, (ii) examine whether CT-altered regions exhibit corresponding FC changes, and (iii) explore associations between neuroimaging metrics, thyroid hormone levels, and neurocognitive performance. By integrating structural and functional measures with detailed cognitive assessment, this work seeks to clarify the neurobiological correlates of cognitive impairment in SHypo and to provide a more comprehensive brain-based characterization of this common endocrine condition.

## Methods

### Participants

This study involved 27 SHypo patients (9 males, 18 females) who were recruited between 2019 and 2021. The inclusion criteria were: (1) Patients with serum TSH levels ≥ 4.20 mIU/L, as well as normal FT3 and FT4 levels, at initial laboratory assessment; (2) Right-handedness to control for potential functional lateralization effects in neuroimaging analyses; (3) Patients with ≥ 8 years of formal education; (4) Patients aged < 60 years; and (5) Patients without conditions affecting brain structure or function (e.g., stroke, brain tumors, significant white matter hyperintensities). On the other hand, the exclusion criteria were: (1) Patients with psychiatric or autoimmune diseases; (2) Patients who recently used (within the past 2 months) THs or Glucocorticoids (GCs); (3) Patients with other endocrine disorders; (4) Patients with clinical manifestations of hypothyroidism or neuropsychiatric abnormalities; (5) Patients with brain structure/function conditions; (6) Prior use of psychotropic medication; (7) Illiteracy; and (8) Non-cooperation. Participants were excluded based on psychiatric history and psychotropic medication use, assessed through a clinical interview and medical record review, focusing on current diagnoses or medication use within the past six months.

Meanwhile, 27 Healthy Controls (HCs) (12 males, 15 females) were recruited. These controls were identified through community advertisements and were group-matched to the SHypo patients based on key demographic variables, including age, sex, and years of education. The same exclusion criteria applied to the SHypo patients were also used for the HCs. Additionally, all HCs underwent thyroid function testing to confirm euthyroid status, with TSH levels within the normal reference range. The Ethics Committee of the Affiliated Nanjing Brain Hospital of Nanjing Medical University approved the study protocol, which adhered to the principles of the Declaration of Helsinki. All subjects provided written consent before participating in the study.

### Serum TH level assessments

Thyroid function testing was standardized to minimize measurement variability. All participants fasted for 8 h prior to blood collection, with samples collected within 24 h of MRI completion. TSH, FT3, and FT4 were measured by electrochemiluminescence (Roche Cobas E601) with rigorous quality control. Reference intervals were: TSH 0.27–4.2 mIU/L, FT4 12–22 pmol/L, and FT3 3.1–6.8 pmol/L. The assays demonstrated inter-assay coefficients of variation of 5–9% and intra-assay coefficients of variation of 3–6%, indicating acceptable precision for clinical research. Single measurements of TSH, FT3, and FT4 were used in the subsequent correlation analyses with neuroimaging and cognitive measures.

### Neurocognitive assessment

All participants underwent a comprehensive suite of neurocognitive evaluations to assess cognitive function. The subtests in these evaluations were grouped into four main areas, as in our previous studies: Attention, memory, processing speed, and EF [23]. The Digit Span Forward (DSF) task was used to assess attention, which measures auditory attention span through immediate, ordered-digit recall [24]. Memory function was divided into verbal and visual domains, which were evaluated using the Logical Memory (LM) for story recall and Figural Memory (FM) for visual pattern recognition, components of the Wechsler Memory Scale-Revised (WMS-R) [25]. Processing speed was assessed using the Digit Symbol Substitution Test (DSST) [26], a task measuring visual-motor processing speed.

On the other hand, EF was assessed using two tests: the Trail Making Test Part B (TMT-B) for cognitive flexibility and the Digit Span Backward (DSB) for working memory [27]. To ensure interrater reliability and minimize variability, all test administrators received standardized training and followed identical administration and scoring protocols. The same team of assessors conducted all evaluations throughout the study period.

### Data acquisition

All MRI data were acquired using a 3T Siemens Magnetom Prisma scanner (Siemens, Erlangen, Germany) equipped with a 32-channel head coil. A T1-weighted, 3D gradient-echo pulse sequence was used for anatomical imaging. First, the participants were advised to remain still during the scan, then placed in a birdcage coil, with soft earplugs to minimize head movement. The T1 mapping MRI settings were: a Repetition Time (TR) of 1900 ms, an Echo Time (TE) of 2.48 ms, and a Flip Angle (FA) of 9°, with 176 axial slices acquired at a slice thickness of 1 mm. These parameters yielded a voxel resolution of 1 × 1 mm^2^ within a Field of View (FOV) of 25 × 25 cm^3^. Resting-state Blood Oxygen Level Dependent (BOLD) MRI was acquired using a standard functional MRI protocol employing gradient-echo and echo-planar imaging. The specific parameters were: TR = 3000 ms; TE = 40 ms; FA = 90°; number of slices = 32, FOV = 24 × 24 cm^2^; slice thickness = 4 mm; slice gap = 4 mm; matrix size = 64 × 64; in plane voxel resolution = 3.75 mm × 3.75 mm; and volumes = 133. A trained neuroradiologist evaluated all images to identify any artifacts or structural irregularities.

### CT analysis

The 3D-T1-weighted sequence images were processed using FreeSurfer (stable version 7.4.1) as detailed in previous research [28, 29]. The process could be summarized as follows: (1) Removal of non-brain tissue; (2) Registration to the Montreal Neurological Institute (MNI) space; (3) Intensity inhomogeneity correction; (4) Tissue type classification; and (5) Probabilistic anatomical labeling.

The CT was computed as the shortest distance between the pial surface and the Gray Matter /White Matter (GM/WM) boundary at each point across the cortical mantle. All cortical reconstructions were visually inspected and, when necessary, manually corrected by a trained researcher following the FreeSurfer quality control and editing guidelines, including inspection of skull stripping, WM segmentation, and pial surface placement, as recommended in the FreeSurfer manual and prior publications [28, 30], before statistical analysis.

### FC analysis

First, all functional imaging data were processed using Statistical Parametric Mapping 12 (SPM12) and RESTplus [31] in MATLAB. The initial ten functional volumes were then discarded to stabilize the scanner and acclimate the participant. Subsequently, slice timing, head-motion correction, and spatial normalization to the Montreal Neurological Institute (MNI) template with a resampled voxel size of 3 × 3 × 3 mm³ were performed. Data with head movement > 2 mm or > 2° were removed before analysis to prevent movement-related bias. Additionally, several spurious variances—including Friston 24 head motion parameters, Cerebrospinal Fluid (CSF) and WM signals, and linear trends—were regressed out. Frame-wise displacement was computed using motion correction-obtained translational and rotational displacements. All images were normalized to the MNI template, and no participants were excluded due to excessive head motion. To further control for potential confounding effects of micromovements in group-level analyses, mean framewise displacement (FD) was included as a nuisance covariate in all functional connectivity group comparisons. The normalized data were spatially smoothed with a 6-mm Gaussian kernel and then linearly detrended and temporally band-pass filtered (0.01–0.08 Hz) to remove noise. Brain regions with significant intergroup differences in CT were designated as seed regions for seed-to-voxel FC analyses. The seed region’s mean time series was determined using Restplus software. This seed time series and the time series of each voxel throughout the entire brain were then subjected to Pearson correlation analysis, with the resulting correlation coefficients (*r*) transformed using Fisher’s z transformation to generate FC maps for each participant.

### Statistical analysis

Statistical analyses of demographic, hormonal, and neurocognitive data were performed using SPSS 21.0 (IBM Corp., Armonk, NY, USA). Data normality was assessed using the skewness-kurtosis test. Normally and non-normally distributed data were expressed as mean ± SD and medians (IQR), respectively. On the other hand, categorical variables were presented as counts (N) and percentages (%). Intergroup comparisons were performed using two-sample *t*-tests, nonparametric tests, and chi-square analyses to examine differences in demographic, TH, and cognitive function test scores. Results with *p* < 0.05 were considered statistically significant.

For the neuroimaging analyses, group comparisons of cortical thickness (CT) across the entire brain were performed using FreeSurfer (v7.4.1). Specifically, CT maps of the left and right hemispheres were separately projected onto the FreeSurfer “faverage” surface and smoothed using a Gaussian kernel with a Full Width at Half Maximum (FWHM) value of 10 mm. Statistical significance levels were set at *p* < 0.001 and *p* < 0.05 for vertex- and cluster-level analyses, respectively. Cluster-level correction for multiple comparisons was conducted using permutation simulations with 5,000 iterations.

Functional connectivity (FC) analyses were conducted using SPM12 and RESTplus. In the subsequent rsFC analysis, statistically significant CT clusters were utilized as seed regions. The rsFC maps were converted to *Z* maps through Fisher’s *Z* transformation and compared across groups. Multiple comparisons were corrected using the nonstationary cluster-level Family-Wise Error (FWE) method, yielding cluster-defining and corrected cluster significance thresholds of *p* = 0.001 and *p* < 0.05, respectively.

We further conducted a partial correlation analysis to examine the connection between TH levels, cortical measurements, and cognitive assessments, while controlling for age, gender, and education. The analysis was conducted separately for the two groups to allow for between-group comparisons. Furthermore, this analysis was performed without correction for multiple comparisons to maintain an exploratory approach aimed at identifying potential links.

## Results

### Demographic, TH, and neurocognitive tests

As shown in Table 1, the SHypo and healthy control groups were well-matched on demographic variables, including age (*p* = 0.695), gender distribution (*p* = 0.402), education level (*p* = 0.712), and marital status (*p* = 0.785). As expected, SHypo patients showed significantly elevated TSH levels compared to controls (*p* < 0.001). In contrast, FT3 and FT4 levels were comparable between groups (*p* = 0.560 and *p* = 0.679, respectively). In cognitive testing, SHypo patients demonstrated specific deficits in visual memory, as measured by WMS-FM (*p* = 0.002), and in cognitive flexibility, as assessed by TMT-B (*p* = 0.019). In contrast, other cognitive domains, including attention, processing speed, verbal memory, and working memory, showed no significant group differences (*p* < 0.05, Table 2).

**Table 1:** Sociodemographic and clinical characteristics and results of the neuropsychological assessment of the subclinical hypothyroidism and healthy controls

	SHypo (*n* = 27)	HC (*n* = 27)	t / χ2 / Z	*P*
**Demographical and clinical measures**				
Age, years	30.22 ± 6.74	29.41 ± 8.38	0.394	0.695 (*t*)
Female gender, n (%)	18 (66.7%)	15 (55.6%)	-0.701	0.402 (*c*)
Education, years	12.41 ± 2.93	12.11 ± 2.94	0.371	0.712 (*t*)
Married, n (%)	13 (48.1%)	14 (51.9%)	-0.074	0.785 (*c*)
**Serum concentrations**				
FT3 (pmol/L)	4.78 ± 0.67	4.69 ± 0.47	0.587	0.560 (*t*)
FT4 (pmol/L)	16.05 ± 2.01	16.28 ± 1.91	-0.416	0.679 (*t*)
TSH (mIU/L)	5.92 (4.51–9.10)	2.23 (1.45–2.82)	3.674	< 0.001^**^ (*Z*)
**Cognitive Function**				
**Attention**				
Digit Span Forward	9.37 ± 1.21	9.91 ± 1.23	-1.618	0.112 (*t*)
**Processing speed**				
DSST	52.44 ± 8.10	53.49 ± 14.36	-0.327	0.745 (*t*)
**Visual Memory**				
WMS-FM	15.30 ± 2.52	17.22 ± 1.78	-3.238	0.002^**^ (*t*)
**Logical Memory**				
WMS-LM	10.26 ± 2.50	14.96 ± 1.40	-1.206	0.233 (*t*)
**Cognitive Flexibility**				
TMT-B	47.94 ± 10.07	42.45 ± 6.13	2.422	0.019^**^ (*t*)
**Working Memory**				
Digit Span Backward	6.74 ± 1.35	7.33 ± 1.49	-1.531	0.132 (*t*)

Note: SHypo: subclinical hypothyroidism; HC: Healthy Controls; FT3, Free Triiodothyronine; FT4, Free Thyroxine; TSH, Thyroid-stimulating Hormone; Comparisons were conducted using *t*-tests (*t*), Kolmogorov-Smirnov tests (*z*), and chi-squared tests (*c*)

* *p* < 0.05; ** *p* < 0.001

**Table 2:** Regions where CT differs between SHypo patients and HC after controlling for age and sex

Region:	Hemisphere	Cluster size	MNI coordinates			CWP*
		(mm^2^)	x	y	Z	
RMFG	L	404.89	-17.6	61.6	1.4	0.01594
precuneus	L	271.11	-15.5	-45.2	47.9	0.04742
CMFG	L	374.16	-35.1	17.6	27.9	0.01792
SFG	L	374.35	-21.3	13.3	50.8	0.01792
ICC	L	925.97	-5.4	-42.9	31.4	0.00200
cuneus	R	265.30	7.0	-79.8	30.7	0.04547
PCC	R	258.42	12.9	-29.4	36.8	0.04742

ALL regions show cortical thinning in SHypo patients

*Clusterwise *p*-value or *p*-value of the cluster

**Fig. 1:**
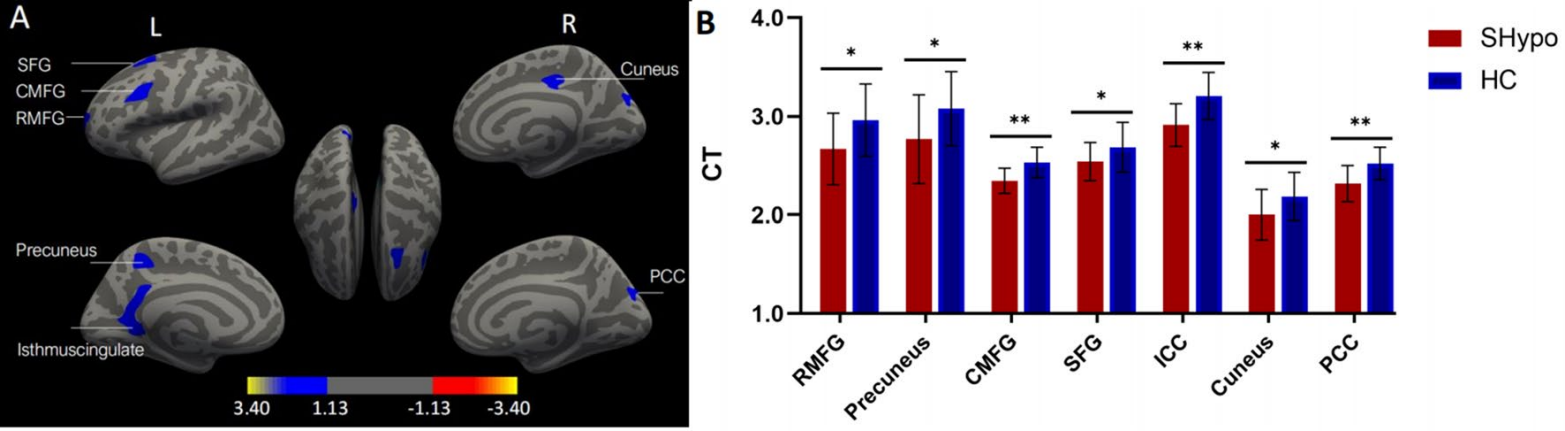
**(A)** Cortical clusters with significantly lower CT values in SHypo patients than HCs (CWP < 0.05). **(B)** The group differences in CT values are illustrated by representative bar plots. SHypo, subclinical hypothyroidism; HC, healthy control; CT, cortex thickness; L, left; R, right; RMFG, rostral middle frontal gyrus; CMFG, caudal middle frontal gyrus; SFG, superior frontal gyrus; ICC, isthmus cingulate cortex; PCC, posterior cingulate cortex. CWP, clusterwise probability. *p < 0.05; **p < 0.001

Regarding the structural MRI results, SHypo patients exhibited lower CT values in the left Isthmus Cingulate Cortex (ICC), Rostral Middle Frontal Gyrus (RMFG), Superior Frontal Gyrus (SFG), Caudal Middle Frontal Gyrus (CMFG), precuneus, right cuneus, and Posterior Cingulate Cortex (PCC). The significance levels for the peak and cluster were set at *p* < 0.001 and *p* < 0.05, respectively (Fig. 1; Table 2). Post-hoc analyses indicated that these structural differences were associated with large effect sizes (Cohen’s d), particularly in the precuneus (d = 1.47), left isthmus cingulate (d = 1.32), and caudal middle frontal gyrus (d = 1.30). Our sample size is comparable to those in previous neuroimaging studies of SHypo that have reported robust structural alteration [14, 32].

Functional connectivity analyses were performed using all seven cortical thinning regions as seeds. Of these, only the left ICC seed revealed significant between-group differences in FC. In particular, SHypo patients showed lower FC values between the left ICC and the right Middle Occipital Gyrus (MOG) (FWE-corrected; Table 2; Fig. 1). The remaining six seed-based FC analyses did not yield any significant results after multiple-comparison correction.

**Fig. 2:**
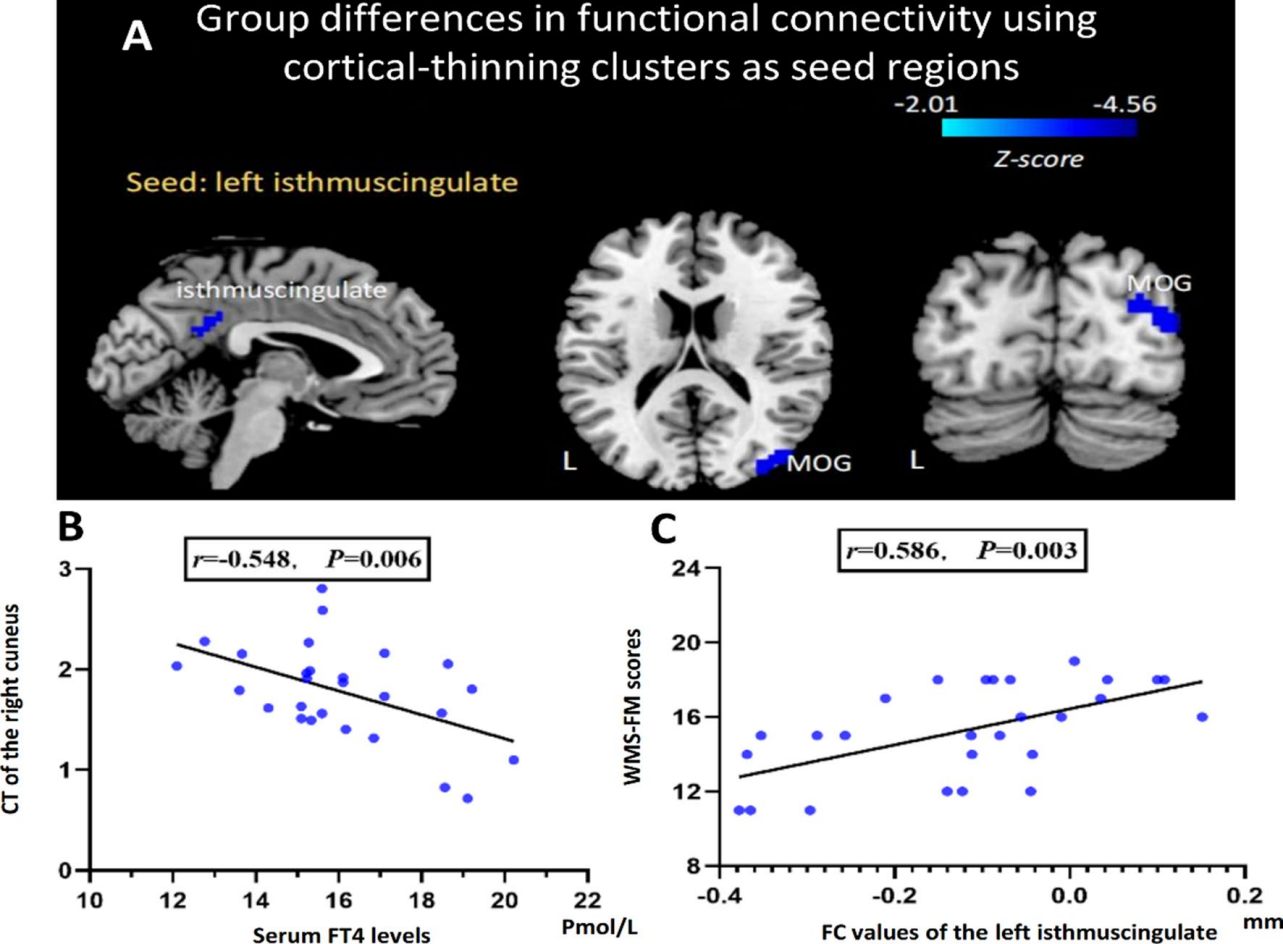
Group differences in functional connectivity using clusters of cortical thinning as seed regions. All clusters show reduced FC in SHypo patients **(A)**, The partial correlation between serum FT4 levels (X-axis) and the CT of the right cuneus (Y-axis) in SHypo patients **(B)**, The partial correlation between the FC values of the left isthmuscingulate and the right MOG (X-axis) and the WMS-FM scores (Y-axis) in SHypo patients **(C)**. SHypo, subclinical hypothyroidism; L, left; MOG, middle occipital gyrus; CT, cortex thickness; FC, functional connectivity; WMS-FM, Wechsler Memory Scale-figural memory

Moreover, in SHypo patients, serum FT4 levels correlated negatively with the CT of the right cuneus (*r* = -0.548, *p* = 0.006; Fig. 2A). Meanwhile, the FC between the left ICC and the right MOG also correlated positively with WMS-FM scores (*r* = 0.586, *p* = 0.003; Fig. 2B). In the healthy control group, neither the correlation between FT4 levels and cuneus CT (*r* = 0.229, *p* = 0.281) nor the correlation between left ICC-right MOG functional connectivity and visual memory scores (*r* = 0.223, *p* = 0.292) reached statistical significance.

## Discussion

This study employed an integrated approach combining structural MRI and rs-fMRI analyses to investigate structural and functional brain changes in SHypo patients, with particular focus on their association with cognitive decline. As a cross-sectional investigation, this study identifies associations. However, it cannot establish causal relationships between SHypo and the observed neural alterations. Notably, SHypo patients exhibited significant cortical thinning in the prefrontal and limbic regions. Additionally, we observed altered functional connectivity between the left isthmus cingulate cortex (a node of the default mode network) and the right middle occipital gyrus (a visual processing region).

These functional alterations, along with the observed cognitive deficits, suggest a network-level dysfunction. Specifically, the FC between the left ICC and right MOG correlated with visual memory scores. At the same time, cortical thinning did not show a direct correlation with cognitive measures. Moreover, FT4 levels correlated negatively with CT, even within the normal TSH range, suggesting that subtle TH variations may be associated with neural changes in SHypo patients. Although FT4 levels were within the normal range in our SHypo cohort, the correlation with cuneus structure suggests that even subtle, within-person variations in thyroid hormone bioavailability may exert a measurable influence on brain structure.

Our findings add to the existing literature on the neurobiological effects of SHypo, particularly regarding cortical thinning and altered brain function. Overt and subclinical hypothyroidism were previously linked to structural brain changes, including GM reductions in regions such as the PFC and hippocampus—areas critical for memory and cognitive flexibility [18, 33, 34]. These findings illuminate the pathophysiological mechanisms of SHypo, particularly in relation to potential early-stage neurodegeneration in regions critical for cognitive processes. Herein, we discovered a cortical thinning pattern—primarily localized to prefrontal cortices, cingulate regions, and the precuneus. The precuneus and posterior cingulate are key nodes of the default mode network (DMN)—a large-scale brain network associated with internal mentation and memory processes [35]. The involvement of these DMN-related regions provides a potential anatomical substrate for the cognitive alterations observed in SHypo patients. We also noted deficits in memory and cognitive flexibility tests, indicating that the above-mentioned disruptions in brain networks align with the cognitive impairments observed in SHypo patients. Collectively, these findings enhance our understanding of SHypo’s impact on brain health. Overall, subclinical thyroid dysfunction may be associated with cognitive-impairment-related neural changes, with a more profound neurobiological impact than previously recognized.

Our findings further revealed that serum FT4 levels correlated significantly with CT in the right cuneus—a visual association hub —suggesting that TH levels could exert region-specific neurotrophic effects on posterior cortical networks. Mechanistically, the cuneus’s reliance on TH-regulated glucose metabolism [36, 37] and angiogenesis [2, 38] could make it vulnerable to even subtle hormonal fluctuations—especially given its high metabolic demands for visuospatial processing. A high expression of Thyroid Receptor Alpha-1 (TRα1) has previously been reported in the occipital cortex [39, 40], where FT4 may activate nuclear pathways that promote neuronal survival and synaptic plasticity [41], thus supporting the aforementioned structural-hormonal link. Moreover, consistent with previous rs-fMRI evidence, which showed reduced cuneus connectivity in experimentally induced SHypo [42], our findings further highlighted the cuneus as a sentinel region for thyroid-related neural compromises.

The observed FC alteration involved a key DMN node (the isthmus cingulate) connecting to a visual processing region, and this alteration correlated with visual memory performance [1, 43]. Previous research suggests that THs could modulate DMN integrity via Brain-Derived Neurotrophic Factor (BDNF)-mediated synaptic plasticity [44] and vascular coupling mechanisms [45], with FC exhibiting greater sensitivity to hormonal changes. The pattern of posterior cortical and cingulate involvement shows some topographic overlap with regions affected in various metabolic and neurological conditions, suggesting these regions may represent vulnerable hubs in the brain [46, 47]. However, whether SHypo shares specific mechanistic pathways with neurodegenerative conditions remains to be directly investigated.

Despite its valuable insights, this study has some limitations. First, methodological considerations should be noted. The modest sample size, while comparable to previous pioneering studies, may limit the generalizability of the findings and preclude meaningful sex-specific or subgroup analyses. Furthermore, the cross-sectional design prevents causal inference regarding the relationship between SHypo and the observed brain changes. Second, several potential confounding factors could not be fully addressed. We lacked data on body mass index (BMI). We did not systematically assess subclinical symptoms of depression or sleep disorders using standardized scales. Given the high prevalence of these conditions in SHypo and their independent associations with brain structure and function, their potential influence on our results cannot be ruled out.

Additionally, the relatively standard spatial and temporal resolution of our rs-fMRI sequence may limit the sensitivity to detect subtle functional alterations. Second, due to the study’s cross-sectional design, it remains unclear whether the observed cortical morphological abnormalities were a direct result of SHypo pathology or represented a pre-existing condition. Therefore, longitudinal research is required to examine further the temporal relationship between TH levels and brain structural changes. Furthermore, the cognitive assessment was not exhaustive; it did not comprehensively assess all subdomains of executive function (e.g., inhibition, planning). Also, it omitted other important domains such as language and visuospatial abilities, which preclude interpretation of the functional significance of the right cuneus finding.

Moreover, the reported correlations were exploratory and not corrected for multiple comparisons, and thus should be interpreted with caution. Additionally, while participants with formal psychiatric diagnoses were excluded, we did not employ standardized scales to quantify subclinical symptoms of anxiety or depression. These subclinical symptoms could potentially influence cognitive test performance and brain measures, and their exclusion represents a limitation of the current study. Finally, we could not account for autoimmune thyroid disease (AITD) due to a lack of antibody testing, which may confound the association between thyroid function and the observed brain changes. To gain a more comprehensive understanding of the relationship between SHypo and brain health, future research will be required to address these limitations.

## Conclusion

This study highlights the neurobiological impact of SHypo on brain structure and function, particularly in relation to cognitive decline. Specifically, we found that even subtle TH fluctuations could induce early brain alterations, highlighting the need for early detection and intervention. These results offer not only valuable insights into SHypo’s potential role in neurodegeneration but also a foundation for future research aimed at improving clinical outcomes.

## Data Availability

All raw data and code are available upon request.
